# Dendritic cell activation and screening for key molecular signatures required for the induction of allergic responses

**DOI:** 10.5339/qmj.2022.fqac.15

**Published:** 2022-04-18

**Authors:** Mohammad Ameen Al-Aghbar, Tracy Augustine, Meritxell Espino Guarch, Rana El Nahas, Ghalia Missous, Nicholas van Panhuys

**Affiliations:** ^1^Laboratory of Immunoregulation, Sidra Medicine, Doha, Qatar E-mail: nvanpanhuys@sidra.org

**Keywords:** allergic response, DC activation, molecular signature

## Abstract

The chain of events that leads to the sensitization of the immune system to environmental antigens, resulting in the onset of allergic disease, has been studied in great detail over the past 30 years. However, during this time, the rate of allergic diseases has increased exponentially, indicating the need to concentrate our studies on host-environmental factors that contribute to the onset of disease. Monocyte-derived dendritic cells (DCs) play a key role in driving localized and systemic immune responses. In this study, we developed a platform for screening the molecular signature and phenotypic profile of DCs activated by allergenic stimuli, including TSLP, IL-25, IL-33, IL-1a, Vit-D3 (1α,25-Dihydroxyvitamin D3), PAR1-AP Peptide, Papain, and recombinant human DerP1 protein to induce a type II associated inflammatory signature. Following activation with allergenic stimuli, modulated DCs are subjected to deep phenotyping via flow cytometry for surface and intracellular markers to detect and/or validate immunomodulatory properties. RNA sequencing is further used to compare the gene expression profiles of DCs responding to either allergenic or microbial stimuli, including the TLR3 agonist dsRNA Poly I:C and TLR4 agonist LPS. In our study, we aimed to identify key molecular signatures of DCs involved in the development of asthma and allergy based on their comparative activation with this broad panel of allergens. We expect to determine central control modules of transcription factors in DCs associated with Th2 induction.

## Figures and Tables

**Figure 1.**
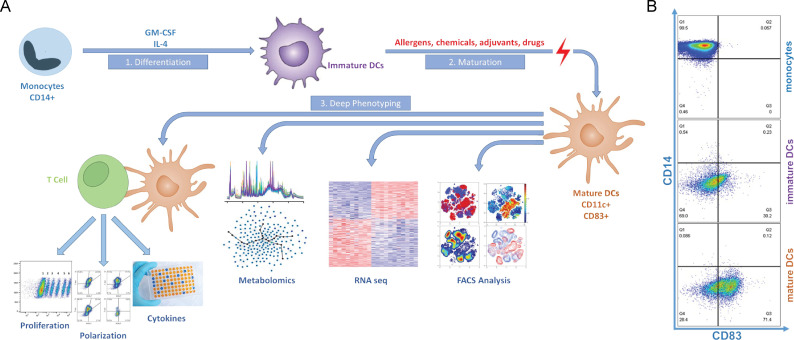
DC Screening Platform. **A.** Isolated monocytes are differentiated to immature DCs, then a panel of allergens are added to get the mature DCs. mDCs are characterized by deep phenotyping using FACS analysis, RNA seq, and metabolomic profile. Immune response analysis is measured by the impact on T cells by direct DC-T cell interaction. Activated T cells will be analyzed for proliferation, polarization, and cytokine secretion. **B.** FACS analysis of monocytes, imDCs and mDCs for CD14 and CD83 surface expression.

